# Shaping the Therapist, Shaping the Field: A Narrative Review of the Person‐of‐the‐Therapist Training (POTT) Approach

**DOI:** 10.1111/jmft.70161

**Published:** 2026-08-03

**Authors:** Sezercan Ucar, Jody Russon, Alba Niño

**Affiliations:** ^1^ Department of Human Development and Family Science Virginia Tech Blacksburg Virginia USA; ^2^ Marital and Family Therapy Program Alliant University San Diego California USA

**Keywords:** marriage and family therapy, narrative review, person‐of‐the‐therapist training, self of the therapist, therapist training, use of self in therapy

## Abstract

The Person‐of‐the‐Therapist Training approach is a relational framework that supports therapists in intentionally integrating personal experience into clinical work. Despite its growing presence in couple/marriage and family therapy training, no comprehensive synthesis has traced POTT's historical development, empirical foundations, and contemporary relevance. This narrative review employed a structured literature search and thematic synthesis of 30 sources published between 1987 and 2024. Three developmental periods are described: philosophical foundations emphasizing therapist personhood, institutionalized training with emerging qualitative evidence, and recent global, sociocultural, and supervisory expansions. Across three periods, the literature reflects increasing conceptual clarity, pedagogical structure, and attention to ethical and embodied therapeutic presence. Implications include the need for increasing the transportability of POTT, expanding its empirical base, and employing it in diverse supervision contexts. In an era of technological mediation and artificial relationality, POTT clarifies the importance of the embodied, ethical, and human‐centered psychotherapy dimensions.

## 1 Introduction

The person‐of‐the‐therapist training (POTT) framework is designed to help therapists intentionally and skillfully bring their personal selves into the therapeutic space. The goal of POTT is to promote therapists' effectiveness by embracing all aspects of self with purpose and awareness (Aponte and Kissil 2016). While most couple/marriage family therapy (C/MFT) programs integrate training devoted to self‐awareness into their curricula, few have systematically adopted structured efforts to train therapists on effective use of self (King and Russon 2023). The POTT model has shown its ability to enhance clinical training, particularly for therapy trainees who are being introduced to the field (Lutz and Spell Irizarry 2009). Indeed, POTT guides emerging professionals toward a deeper understanding and mastery of themselves, thereby strengthening their ability to connect, assess, and intervene with clients (Aponte et al. 2009). At the heart of this training is the human‐centered belief that a therapist's personal life experiences, cultural backgrounds, and worldviews are integral to their professional role and essential to practicing in ways that are both professional and deeply personal. Accordingly, a central premise in POTT is that all therapists are *wounded healers* (Nouwen 1979).

### 1.1 The Person of the Developer

The person behind POTT is Harry J. Aponte, MSW, LCSW, LMFT. Raised in New York City by Puerto Rican parents, Aponte has brought a rich blend of personal experience, cultural understanding, and deep compassion to his work with diverse families and communities. Colleagues and scholars have described him as a clinician‐teacher whose humanity, humility, and relational presence were central to both his practice and mentorship (Niño and Zeytinoglu‐Saydam 2023). As a central figure in the field of C/MFT, Aponte is known for his leadership at the Philadelphia Child Guidance Clinic and for integrating culture and spirituality into clinical practice (LaRiviere and Robertson 2019). Aponte describes his work as integrative. Although he worked closely with Salvador Minuchin to implement and expand structural family therapy (Aponte and Kissil 2015; Aponte and VanDeusen 1981), his professional background is also grounded in the psychoanalytic tradition. Consequently, the POTT approach reflects Aponte's thoughtful integration of distinct therapeutic traditions. He describes the development of POTT, in part, as a reaction to the purist application of both structural family therapy and traditional psychoanalysis (Aponte 1992; Niño and Zeytinoglu‐Saydam 2023).

From the early 2000s to the early 2020s, Aponte served as a Clinical Associate Professor in the C/MFT program at Drexel University, while also maintaining a private practice and providing clinical supervision and consultation in Philadelphia. Although the POTT approach did not originate in an academic environment, it served as an integral component of training for master's students at Drexel University. In this academic program, Aponte adapted the POTT approach to train first‐year C/MFT students (Aponte et al. 2009). Then, using the knowledge gained from embedding POTT into an academic curriculum, he co‐edited the book, *The Person of the Therapist Training Model: Mastering the Use of Self* (Aponte and Kissil 2016), to serve as a guiding resource for students, instructors, and supervisors. Due to his contributions to the field of family therapy, Aponte has received numerous honors and awards, including the 1992 Distinguished Contribution to Family Therapy and Practice Award from the American Family Therapy Academy, 2001 Outstanding Contribution to Marriage and Family Therapy Award from the American Association for Marriage and Family Therapy, 2004 Degree of Doctor of Humane Letters from Drexel University, and 2006 Degree of Doctor of Public Service from University of Maryland.

### 1.2 Overview of the Approach

Over time, Aponte has translated the concept of the *wounded healer* into a systematic training strategy to help therapists access and apply their lived experience in ways that enable more attuned and effective clinical practice. True to his belief that therapy should be an experience, through his scholarship, teaching, and supervision Aponte has encouraged therapists to consider how they, themselves, in addition to their chosen theory or model, can contribute to core therapeutic elements: relationship‐building, assessment, and intervention (Aponte and Kissil 2016). Core components of POTT help therapists remain present by cultivating *self‐awareness*, *accessing the self* in session, and learning to work deliberately with clients through *self‐management*. When training in these three components, the ongoing practice of *identification* and *differentiation* is centralized in therapeutic encounters (Aponte and Ingram 2018). Indeed, POTT emphasizes how identification (experiencing with and from clients' perspectives) and differentiation (recognizing the differences between oneself and clients) must work in tandem (Aponte and Ingram 2018). As one of the few frameworks that offer a structured pathway for the intentional integration of therapists' personal selves into clinical work (Aponte and Carlsen 2009), POTT and its components have been incorporated into graduate training and clinical supervision. Recent scholarship has also situated the model within broader conversations on therapist factors and therapist development (e.g., Middleton et al. 2024). Yet, no review to date has synthesized the empirical POTT literature, its historical evolution, and emerging future directions. The present narrative review addresses this gap by tracing POTT's development across three distinct periods, synthesizing thematic patterns in the literature, and identifying implications for training, supervision, ethics, and contemporary clinical practice.

## 2 Methods

To examine the evolution of the POTT framework, this paper employed a narrative review design (Green et al. 2006; Sukhera 2022) with a structured literature search and thematic synthesis. The narrative approach enabled historical mapping, conceptual integration, and interpretive synthesis of a literature base that is theoretically rich, methodologically diverse, and primarily qualitative.

### 2.1 Search Strategy and Selection Criteria

A comprehensive literature search was conducted using PsycINFO and Family and Society Studies Worldwide, supplemented by citation tracking of foundational POTT publications. Initial database searches were followed by iterative reference list reviews and forward citation searches to identify additional relevant sources. These were the complete set of search terms used in the database searches and were combined in varying configurations: *person of the therapist, self of the therapist, use of self, therapist self, therapist personhood, therapist development, therapist training, training, supervision, clinical supervision*, and *Harry Aponte*. Citation tracking was used to identify influential works that may not have been captured through database indexing alone, particularly early conceptual and book‐based contributions. Source identification, screening, and inclusion decisions were discussed collaboratively by the first two authors throughout the review process.

**Figure A1:**
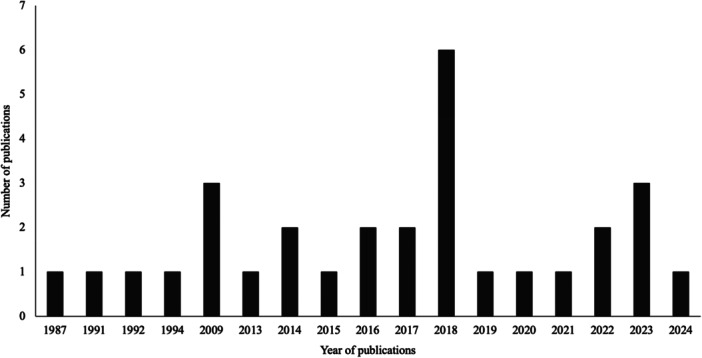
Distribution of publications on the POTT approach by year. [Color figure can be viewed at wileyonlinelibrary.com]

To be included, sources were required to meet the following criteria: (a) to explicitly define, apply, theorize, or empirically examine the POTT approach as developed by Harry Aponte; (b) to focus on training, supervision, or therapist development processes; and (c) to have been published between 1987 and 2024, reflecting the period from the model's earliest conceptualizations to its most recent global adaptations. The final corpus comprised 30 sources (see Figure A1), including 15 peer‐reviewed theoretical or conceptual articles, 10 peer‐reviewed empirical studies, and five books or book chapters.

Empirical studies included in the review employed exclusively qualitative methodologies, including grounded theory, reflexive thematic analysis, phenomenological approaches, and autoethnography. No quantitative evaluations of POTT training outcomes were identified. This absence reflects an important limitation in the current evidence base and highlights the need for future methodological pluralism in evaluating POTT processes and outcomes. Notably, much of the existing empirical literature has been produced by scholars who were trained within or closely connected to the Drexel University C/MFT program, where the POTT model was originally developed and implemented. Many authors contributing to this body of work completed their graduate training within this context and/or participated directly in POTT training (e.g., Kissil, Niño, Zeytinoglu‐Saydam, Russon, Pennant, Apolinar Claudio) and worked closely with Aponte. As a result, the empirical literature largely reflects scholarship emerging from within the model's originating training community, a pattern that is not unique to POTT and has also been observed in the development of other psychotherapy models. These factors informed the selection of a narrative review approach rather than a systematic or meta‐analytic design.

### 2.2 Analytic Approach

**Figure A2:**
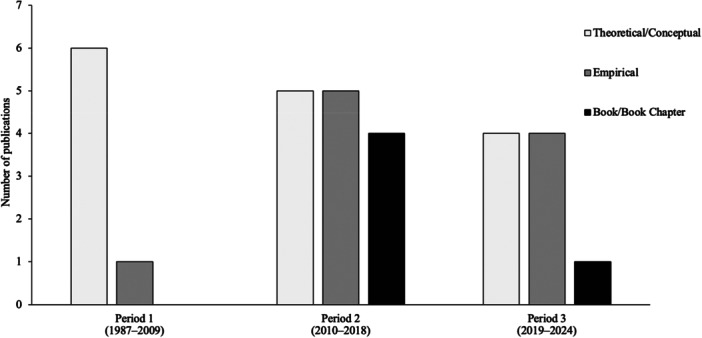
Publication types in POTT literature across periods. [Color figure can be viewed at wileyonlinelibrary.com]

To trace the development of the POTT model, sources were grouped by year of publication and type of contribution (theoretical/conceptual or empirical). This temporal and thematic organization revealed three distinct periods, shaped by the model's evolving focus and applications. These periods were not predefined nor inherent in the literature. Instead, they were constructed through our analytic engagement with the sources. Through an iterative process of reading, comparison, and interpretation, we identified shifts in the focus, language, cultural context, and application of the model. During this process, the first author created analytic notes to track recurring concepts, publication patterns, methodological features, and shifts in how POTT was conceptualized across the literature. These notes were discussed with the second author and used to refine the grouping of sources into developmental periods and subthemes. The subheadings were developed through iterative engagement with the literature rather than through a formal content analysis or a priori coding framework. Together, these reflections were organized into three analytically and chronologically meaningful phases (see Figure A2).

A thematic narrative synthesis was conducted within and across three time periods, attending to four recurring domains: conceptual contributions, training structure and pedagogy, core therapist processes, and reported trainee impacts. These domains emerged inductively as organizing patterns across the reviewed sources. Rather than aggregating findings, this synthesis emphasized the evolution of ideas, assumptions, and applications over time, thereby enabling comparisons across periods and identifying developmental continuities and conceptual turning points.

## 3 Three Periods of POTT Development

### 3.1 Period 1: Foundation and Conceptualization (1987–2009)

The foundational period of the POTT approach was marked by the emergence of a relational philosophy that centered the therapist's person as a vital clinical tool. This period proposed that personal experience could serve as a bridge to therapeutic connection. While the specific applications of the approach were not yet formalized, this period in the evolution of POTT laid the emotional, personal, and conceptual groundwork for what would later become a systematic training model.

#### 3.1.1 Theoretical and Philosophical Roots

The earliest iteration of the POTT approach emerged from a philosophical commitment to the idea that suffering is not a hindrance but a therapeutic resource. Aponte's writings during this period were heavily influenced by Henri Nouwen's (1979) concept of the *wounded healer*, which views the person's pain as a facilitator of connection and presence in the context of clergy. This framing guided the shift from a technique‐focused view of therapy toward an approach that punctuates the therapist's person as an asset. Further, the approach challenged the view that therapists must resolve personal struggles to be effective. Instead, the clinical space was presented as an opportunity for authentic connection *through* the therapists' woundedness. In their formative article, Winter and Aponte (1987) challenged the therapists' emphasis on neutrality and expertise. They argued for what they called the *Person/Practice model*, which suggested that therapists must attend to both internal and external aspects of their development. They introduced four domains of functioning (technical, theoretical, collaborative, and internal) equally necessary to clinical effectiveness. The “internal” domain, defined as the therapist's emotional history and self‐awareness, became the most distinctive area that POTT would later seek to further theorize and operationalize.

Later, Aponte (1992) extended this discussion by examining how person‐of‐the‐therapist work could be integrated into the training of structural family therapists. Rather than treating therapists' personal reactions as distractions from technique, he argued that emotional responses arising during clinical encounters, particularly during live enactments, could serve as important guides for understanding family dynamics and informing intervention. Within this framework, the therapist's internal experience becomes part of the training process, helping clinicians develop greater empathy, awareness, and intentionality in their participation within family transactions.

The operationalization of the internal domain was also extended in Aponte's work on training therapists to work with poor and minoritized families (1991). In this formulation, therapist personhood was understood not only in intrapsychic or relational terms but also within broader social, cultural, and economic contexts. Per this work, therapists inevitably bring their personal histories, social locations, and emotional responses into therapeutic encounters, particularly when working across differences in race, class, and life experience. Thus, training must address the personal, social, and clinical dimensions simultaneously to prepare therapists for active, emotionally engaged work with underserved families and communities (Aponte 1991). This emphasis also reflects Minuchin's broader structural family therapy writings, which situated clinical work with poor and minoritized families within social, cultural, and economic contexts rather than viewing family problems as isolated from structural conditions (Minuchin et al. 1967).

#### 3.1.2 Early Training Practices and Conceptual Language

As POTT began taking shape, one of its most enduring contributions was the concept of the *signature theme*. Aponte introduced this concept as one way to operationalize the therapist's internal domain, conceptualizing it as a recurring pattern in the therapist's own life that shapes how therapists relate to their clients (Aponte et al. 2009). The signature theme concept was still emerging in the early 1990s, and it was formally introduced in Aponte and Carlsen's (2009) writing on supervision, as described in the paragraphs below. The model's early writings, however, made clear that the patterns embedded in therapists' own lives were central to understanding and managing therapeutic relationships.

Incorporating personal material into supervision and classroom training raised important ethical questions as the POTT approach continued to evolve. Aponte (1994a) addressed the ethics of therapists' personal exploration in training directly by distinguishing between dual relationships and dual qualities. While *dual relationships* (e.g., a supervisor assuming a purely therapeutic role with a trainee) violate ethical boundaries, the concept of *dual qualities* refers to a trainee's capacity to be both a student while also having a self or be a clinician‐in‐training and an emotionally present human being. This clarification defended the legitimacy of using one's personal history and experiences of woundedness in training contexts. At the same time, the distinction between personal exploration in training and psychotherapy has remained a recurring question within POTT trainings, where participants often grapple with how to differentiate reflective professional development from therapeutic work. These early works asserted that personal reflection was *not* therapy, but a form of professional preparation.

#### 3.1.3 Application in Clinical Practice and Emerging Tools

By the late 2000s, POTT began the transition from a primarily philosophical approach to a more systematic and structured model. Aponte and Carlsen (2009) introduced the first iteration of the POTT supervision instrument, which outlined a sequential process to help therapists identify, access, and use their signature themes. This instrument represented a significant step toward systematizing what had previously been an intuitive, supervisor‐led process.

In another article published in the same year, Aponte and his colleagues (2009) described their implementation of the POTT in an educational setting for the purposes of training master's level C/MFT students. Building on the early work that distinguished dual relationships from dual qualities, this paper emphasized that the goal of training was not to resolve emerging therapists' personal struggles but to teach them to engage with these struggles clinically. Doing so enabled students to transform their own painful experiences into authentic therapeutic presence. Thus, this article served as both a description of the POTT philosophy as applied in a master's program and a guide for positioning the training systematically within the broader curricula. Together, these two publications marked POTT's emergence as an organized supervision and training method with a growing potential for replicability and transportability.

#### 3.1.4 Early Reflections From Trainees

Building on the foundational tools and supervisory practices outlined in the late 2000s, early trainee reflections provided an initial glimpse into the impact of the POTT approach on therapists. Although no formal outcome data were collected during this period, qualitative accounts illustrated how the model shaped therapist development. In their reflections on the POTT pilot at Drexel University's C/MFT program, Lutz and Spell Irizarry (2009) described how engaging with their signature themes fostered deeper empathy, enhanced emotional regulation, and increased presence during clinical work. As a result of participating in POTT, these two students reported feeling attuned to their internal experiences and more capable of sustaining connections with clients who were both similar to and different from themselves. With the publication of the 2009 articles, the foundational commitments of the POTT approach were firmly established. What remained unresolved, however, was how these philosophical principles could be systematically taught, studied, and evaluated. These unanswered questions set the stage for the model's next phase of development, marked by empirical expansion and institutionalization.

### 3.2 Period 2: Empirical Expansion and Institutionalization (2010–2018)

The second period of POTT development marked a pivotal shift from conceptual foundations to curriculum refinement and implementation. During this period, the model gained academic recognition, curricular coherence, and a growing empirical base, particularly through its institutionalization at Drexel University's C/MFT program. This period was defined by a collective effort to name, teach, and study the use of self in therapy as a legitimate and necessary component of clinical training. The second period also marked a turning point for the POTT approach, as it expanded from institution‐specific implementation to a broader academic audience. A major milestone was the 2018 *Journal of Family Psychotherapy* (i.e., *International Journal of Systemic Therapy*) special issue devoted entirely to POTT. This special issue, titled *The person of the therapist training (POTT) model: Theory, training and therapist personhood*, brought together theoretical, empirical, and practice‐oriented perspectives to provide a fuller perspective on contemporary training issues in the field of systemic family therapy (Aponte and Watson 2018). This publication affirmed the model's relevance to the field and also provided a scholarly platform for articulating its conceptual and pedagogical contributions to psychotherapy at large. The intention of the special issue was exemplified in the introduction, where Aponte and Watson (2018) highlighted Salvador Minuchin's view that it is the therapist, rather than the technique, who drives change in therapy. This framing positioned the following POTT papers as both an evolution of systemic thinking as well as a challenge to models that de‐prioritize therapist presence. The POTT special issue, therefore, signaled a growing consensus that the therapist's personal and emotional selfhood warranted attention in clinical training.

#### 3.2.1 Curricular Formalization and Branding

As the scholarly attention to POTT grew, so did its integration into formal graduate training. Watson (2018) detailed how POTT was branded as a pillar of Drexel's C/MFT program. Far from being a marketing label, branding in this context referred to the embedding of POTT into the program's philosophy, structure, and identity. At the program, the model was no longer presented as one approach among many, but as a central pathway through which students would learn to become relationally attuned and self‐aware clinicians. This institutional embrace was also grounded in POTT's explicit attention to power, identity, and systemic forces. Specifically, the training did not stop at facilitating insight and awareness at the individual level; it required students to situate themselves in relation to race, gender, class, spirituality, and other social dimensions (Aponte 1994b) through active use of identification and differentiation (described below).

The integration of POTT across coursework, supervision, and clinical training was also documented in a program film produced by Drexel University College of Nursing and Health Professions (2017), which illustrated how POTT principles were interwoven throughout the master's curriculum rather than confined to a single class or supervisory space. In particular, the film demonstrated how students were introduced early to reflective practices (e.g., writing about their signature themes, journaling, engaging in clinical enactments through role‐play) that linked personal history and current struggles to clinical presence, reinforcing POTT as an organizing pedagogical framework rather than an isolated training intervention. At Drexel's C/MFT program, POTT became a flagship model for both technical preparation and professional transformation.

#### 3.2.2 Training Framework and Conceptual Advances

With the curricular embedding of POTT came a growing need to further articulate its theoretical underpinnings in a coherent and transferable form. After several years of delivering POTT in Drexel's C/MFT master's curriculum, Aponte and Kissil (2014) provided a critical conceptual contribution that distinguished the clinical application of POTT from therapist self‐disclosure. Their work reframed therapist pain and emotional struggles as a source of relational access and authenticity that may be clinically useful through multiple pathways, including but not limited to self‐disclosure. Rather than requiring therapists to reveal personal information to clients, POTT emphasizes multiple ways of using the therapist's self in clinical work, including drawing on personal experiences to deepen connection with clients, inform clinical assessment, and guide therapeutic interventions. The authors point to the use of therapists' narratives of their core struggle (i.e., signature themes) as a critical resource. In doing so, Aponte and Kissil (2014) underscored POTT's emphasis on the deliberate and intentional clinical use of personal material.

Two years later, Aponte and Kissil (2016) formalized the application of their conceptual developments by publishing the first POTT training manual. This volume systematized and extended the curriculum described in earlier works by articulating a three‐part developmental framework consisting of knowledge of self, access to self, and management of self. The curriculum was intentionally scaffolded along this pathway, beginning with introspective exercises through which trainees identify, write about, and discuss their signature themes. Subsequent activities focus on helping trainees access these themes in the moment of clinical engagement and ultimately learn to manage and use these personal reactions intentionally within therapeutic work. The manual also included reflective journaling prompts, case‐based guides for clinical role‐plays, and teaching supports such as syllabi and example assignments designed to facilitate implementation across training contexts. This volume provided a concrete scaffold for the training process, transforming conceptual philosophies about therapeutic presence into observable and teachable components.

Alongside the formal articulation of POTT's developmental framework, contemporaneous writings further clarified how the therapeutic use of self becomes operationalized within training processes. Aponte and Kissil (2014) described the use of structured reflection on clinicians' signature themes that shape emotional responses, relational patterns, and therapeutic engagement. Rather than framing professional development as the resolution of personal wounds, this approach emphasized conscious acceptance and intentional clinical use of unresolved experience as a pathway to deeper empathic connection and intervention effectiveness. The signature theme exercise (combining written reflection, mentored dialog, and iterative revision) positioned training and supervision as a relational holding environment in which vulnerability, cultural context, and clinical responsibility could be integrated.

Extending the philosophical writings of Period 1, Period 2 publications also challenged enduring assumptions about the separation between personal and professional selves. Kissil et al. (2018) argued that therapist development within POTT involves holistic transformation rather than the acquisition of discrete emotional insights, emphasizing instead the cultivation of a fluid and ethically‐attuned clinical identity. Within this framing, POTT is not oriented toward resolving the past but toward learning to live and work with its ongoing presence. Therefore, training becomes less a journey toward emotional closure and more a process of developing fluency in contradiction alongside intentional clinical use.

In addition, Aponte and Kissil (2017) further clarified the model's developmental logic by situating POTT within a broader tradition of therapist self‐work while emphasizing that the aim of training is not personal resolution but the intentional and clinically purposeful use of lived experience. Their description of Drexel's C/MFT curriculum highlighted a staged experiential process through which trainees identify signature themes, explore their relational meanings, and translate this awareness into supervised clinical action, reinforcing POTT's consolidation during this period as a coherent training approach. Finally, the three‐part developmental framework described by Aponte and Kissil (2016) was further elaborated through the distinction between *ways of being* and *ways of doing*, originally articulated by Kissil. Ways of being refer to the therapist's internal stance toward clients (e.g., presence, humility, emotional awareness), whereas ways of doing refer to the intentional clinical actions through which this stance is expressed in practice. Following this publication, Apolinar Claudio and Watson (2018) built on this distinction to further clarify how internal awareness cultivated through POTT becomes translated into intentional and relational therapeutic action.

#### 3.2.3 Differentiation as a Component of Relating

This period also marked a growing attention to the role of empathic identification and differentiation, which describe the tension between relating to clients and remaining grounded in one's own experience as a therapist (Aponte and Ingram 2018). Supervision environments became spaces in which therapists were taught to remain emotionally present without becoming caught up, thereby striking a balance between resonance and regulation. In a related contribution, Aponte and Nelson (2018) presented a case study in which POTT supported a White male therapist in building rapport with a Black female client. Their work illustrated how emotional congruence and social location interact, suggesting that similarity is not a prerequisite for connection.

#### 3.2.4 Empirical Studies on POTT's Impact

Empirical studies during this period began to document how POTT shaped trainee development and influenced clinical practice. Niño et al. (2014) conducted a retrospective thematic content analysis of final reflection papers written by first year master's‐level trainees following completion of a 9‐month POTT course. Guided by questions regarding perceived professional growth and the clinical relevance of personal exploration, their analysis identified six domains of reported change, including increased emotional attunement, deeper self‐awareness, strengthened capacity for disciplined vulnerability, and an enhanced ability to link personal activation to intervention choices. In a subsequent publication drawing on an expanded version of this dataset, Niño et al. (2016) examined how trainees understood their therapeutic relationships with clients following participation in POTT. Their findings indicated that participants related their POTT training to the development of qualities widely supported in psychotherapy research as contributing to effective therapeutic alliances and positive clinical outcomes, including empathy, management of countertransference, balanced alliances, positive regard, and a strengthened therapeutic bond (Wampold 2015). These findings also align with broader psychotherapy research identifying therapist empathy, genuineness/congruence, therapeutic alliance, and countertransference management as relational processes associated with client outcomes (Elliott et al. 2018; Flückiger et al. 2018; Hayes et al. 2018; Kolden et al. 2018).

Subsequent qualitative investigations complemented these findings by examining both intrapersonal transformation and the sustained clinical use of personal material. Kissil and Niño (2017) analyzed post‐course reflections from master's level trainees and described shifts toward increased self‐acceptance, emotional steadiness, and relational openness, suggesting implications for therapist resilience and self‐care. In a later study, Kissil and Niño (2018) further explored how professional and personal changes unfolded in tandem, highlighting the reciprocal relationship between therapists' ways of being and ways of doing. Apolinar Claudio and Watson (2018) contributed additional evidence through qualitative interviews with program graduates, demonstrating that intentional use of self, cultivated during POTT training, continued to shape clinical decision‐making, supervisory practices, and moment‐to‐moment therapeutic presence years after program completion. These findings, drawn from a distinct data source from the in‐course reflections examined in earlier studies, provided preliminary evidence of the durability of POTT‐related learning beyond graduate education.

The conceptual refinements, institutional embedding, and emerging empirical evidence of this period established POTT as a teachable, researchable, and socially responsive framework for therapist development. However, it is important to note that the empirical studies conducted during this period were based primarily on small cohorts of trainees who had participated in POTT training within the C/MFT program at Drexel University, where the model was originally developed and implemented by Harry Aponte. As such, the available research reflects the experiences of students who were directly exposed to the model within a single institutional context. Additionally, the existing studies have focused exclusively on trainees' perceptions of their professional development, and no empirical studies have yet examined the impact of POTT training on client outcomes. Although data sources therefore remain limited in scope and setting, the convergence of conceptual and empirical work during this period contributed to the model's growing legitimacy within clinical education. In this way, the second period positioned POTT for subsequent expansion beyond its originating institution.

### 3.3 Period 3: New Voices and Global Reach (2019 –2024)

With the retirement of Aponte in 2024 and the emergence of new scholars, this period reflected a generational transition in which the model's core commitments were both reaffirmed and reinterpreted for use across diverse cultural and institutional contexts. As the POTT approach matured, its third period was marked by continued philosophical positioning alongside global expansion and application in new contexts.

#### 3.3.1 Re‐Centering Therapist Presence and Philosophical Renewal

In his later reflections, Aponte (2022) re‐centered the model on therapist presence, emphasizing that “the self as is” remains the central instrument available in the therapy room. Revisiting the wounded healer tradition, he argued that effective therapy does not require the therapist to be healed, but to be grounded, honest, and emotionally available. This framing reaffirmed POTT's foundational conviction that the therapist's personhood is not separable from clinical work and further linked the model to common factors perspectives in which authenticity, alliance, and empathic presence are central mechanisms of change.

#### 3.3.2 Innovations in Supervision, Ethics, and Training Responsibility

A defining contribution of this period was the extension of POTT into supervision practice and the provision of explicit ethical guidance for working with therapists' personal material. Niño and Zeytinoglu‐Saydam (2020) presented a conceptual framework for applying POTT within clinical supervision. The authors outlined structured supervisory practices designed to help supervisees identify and work with their signature themes while maintaining appropriate emotional containment and professional boundaries. They illustrated how supervisory attunement and boundary clarity within POTT allow personal material to inform clinical reasoning without overwhelming the therapist. Supervisors facilitate this process by guiding supervisees to recognize how their signature themes become activated in clinical encounters and by helping them remain emotionally present with these reactions rather than defensively distancing from them. Then, extending beyond supervision technique and integrating a larger systems perspective, Pennant and Shamoon (2023) advanced a conceptual and practice‐based argument that POTT constitutes an ethical and pedagogical responsibility for shaping clinical training environments. Drawing on social justice‐oriented training contexts, they positioned use‐of‐self work as inseparable from attention to identity, power, and institutional dynamics, thereby reframing POTT as a sustained ethical stance rather than a discrete clinical intervention.

POTT was also applied in the context of empirically supported treatments in systemic family therapy. For example, Zeytinoglu‐Saydam and Niño (2019) illustrated the adaptation of POTT within emotionally focused couple therapy (EFT; Johnson 2019) supervision through applied clinical case material, showing how therapists can remain affectively engaged in high‐intensity relational systems while sustaining grounded differentiation. In addition, POTT principles and modified exercises have been applied in the advanced training curriculum for attachment‐based family therapy (ABFT; Diamond et al. 2014) and have been delivered to ABFT therapists in training for almost 10 years (Russon et al. n.d.). In training activities, therapists are guided in understanding and describing their own attachment themes and narratives in a group format that parallels how therapists engage with youth and their parents in ABFT.

#### 3.3.3 Cross‐Cultural and Global Expansion

This period also marked the translation of POTT into diverse sociocultural contexts, demonstrating both the adaptability of the model and the necessity of contextual sensitivity regarding meanings of self, vulnerability, and relational obligation. Through an autoethnographic analysis grounded in their experiences as faculty of color at Antioch University, Pennant and Shamoon (2022) described POTT as a decolonial pedagogical framework that enables trainees to confront dominant cultural norms surrounding emotional expression, legitimacy, and authority within clinical training environments. Their work positioned engagement with therapist personhood as inseparable from broader structures of identity, power, and institutional belonging, thereby extending POTT beyond intrapersonal development into culturally situated ethical practice. Applying this perspective, Oral et al. (2023), using qualitative analysis of couple therapists' clinical reflections in Turkey, identified processes through which therapists negotiated personal history, professional responsibility, and culturally shaped expectations of intimacy and gender.

In Taiwan, Chen and Hsiung (2021) implemented a POTT‐informed training adaptation with graduate students and examined trainee experiences through qualitative thematic analysis that was attentive to contextual dynamics, such as academic pressure, filial expectations, and face‐saving norms. Subsequent findings (Chen 2024) indicated that culturally responsive adaptations supported measurable growth in emotional openness, vulnerability tolerance, and self‐compassion, which were linked to relational clinical competence. Together, these studies point out that cultural responsiveness is foundational to the sustainability, transportability, and ethical application of POTT across global training environments.

#### 3.3.4 Application of POTT Into Specific Areas of Practice

A further development of this period was the clinical application of POTT to specific presenting issues and areas of practice. Oral et al. (2023) qualitative analysis focused on POTT issues unique to the context of couples work. Findings illustrated how POTT principles function for therapists providing care to couples in present and attuned ways and shaped by sociocultural expectations. In addition, through constructivist grounded theory methodology, King and Russon (2023) examined how therapists with histories of eating disorders (i.e., lived experience) regulated self‐disclosure, managed projection, and intentionally employed personal experience in treatment. Their analysis introduced the dynamic processes with regard to how therapists move between identification with clients and reflective distance as an intentional clinical skill as opposed to an automatic reaction. These applied investigations signaled a shift toward examining therapist embodiment as a series of clinically consequential processes within specialized areas of practice.

## 4 Discussion

Tracing the POTT literature across three developmental periods reveals a coherent historical trajectory marked by deepening philosophical clarity, institutional grounding, and expansion of cultural and clinical applications. Early scholarship established the ethical and relational premise that therapists' personhoods are inseparable from therapeutic work (Winter and Aponte 1987). This premise grounded the model in wounded healer philosophy and emphasized therapists' moment‐by‐moment responses (e.g., emotional activity) as a source of clinical information, with the concept of the therapist's signature theme emerging as the central organizing construct for understanding and using these reactions in therapeutic work (Aponte 1992; Aponte and Winter 2013; Winter and Aponte 1987). The second period translated these philosophical commitments into structured pedagogy, institutional training, and an emerging qualitative empirical base, demonstrating how the model supports growth in self‐awareness, emotional regulation, empathy, and intentional use of personal experience (Kissil and Niño 2017, 2018; Niño et al. 2015, 2016). More recent work has extended POTT into supervision, global cultural contexts, and domain‐specific clinical populations while also seeking to refine the processes associated with the use of self through empirical inquiry (King and Russon 2023; Pennant and Shamoon 2022; Zeytinoglu‐Saydam and Niño 2019).

### 4.1 Synthesis Across Periods

Across eras, several trajectories emerged that describe POTT's development over the past decades. First, philosophical commitments have remained central. Specifically, conceptual refinement has accompanied, rather than followed, application. Second, institutionalization enabled the transformation of therapist personhood from an abstract value into teachable and researchable processes, particularly through the publication of the POTT training manual (Aponte and Kissil 2016). Third, cross‐cultural translation and clinical specialization efforts of Period 3 supported movement beyond the model's originating institution toward broader global relevance. Collectively, these trajectories suggest that POTT has evolved from relational philosophy into a globally relevant framework increasingly positioned for systematic evaluation and dissemination.

### 4.2 Contemporary Relevance of POTT Trajectories of Development

Recent literature positions POTT as uniquely responsive to contemporary ethical concerns in psychotherapy. Scholars have pointed out its relevance for training in empirically supported systemic therapies, for specific presenting problems, and with diverse therapists, suggesting that intentional engagement with therapist personhood strengthens relational attunement in clinical practice (Chen and Hsiung 2021; King and Russon 2023; Zeytinoglu‐Saydam and Niño 2019). Other work highlights POTT's role in helping clinicians explore privilege, marginalization, and cultural identity within clinically grounded reflection (Pennant and Shamoon 2022). These contributions align POTT with broader calls for ethically accountable, culturally responsive, and relationally authentic psychotherapy (McDowell et al. 2022). They also resonate with broader psychotherapy research showing that clinical outcomes are shaped by therapist effects and relational processes as well as by techniques (Sprenkle and Blow 2004; Sprenkle et al. 2009; Wampold 2015) such as therapeutic alliance (Flückiger et al. 2018), empathy (Elliott et al. 2018), genuineness/congruence (Kolden et al. 2018), warmth (Seewald and Rief 2023), and effective management of countertransference (Hayes et al. 2018).

POTT's ethical positioning becomes particularly salient within contemporary sociotechnical contexts. Rapid developments in artificial intelligence and digitally mediated interactions are reshaping assumptions about empathy, responsiveness, and relational connection. As generative AI systems increasingly simulate therapeutic dialog and emotional attunement (Hertlein and Springer 2026), psychotherapy faces questions regarding what constitutes authentic presence and whether it is a necessary factor in promoting human growth. In an era of expanding artificial intimacy, the model's insistence on connection through shared human experiences and intentional use of vulnerability highlights dimensions of therapeutic encounters that cannot be reduced to algorithmic responses.

### 4.3 Implications for Therapist Training, Transportability, and Supervision

The developmental arc of POTT highlights critical questions regarding transportability across training environments. Much of the existing literature is derived from programs with direct access to experienced POTT supervisors, particularly those connected with Drexel University's C/MFT program. As interest in the model expands, a central challenge concerns how POTT‐informed training can be embedded within graduate programs that lack locally trained supervisors or institutional infrastructure. The emergence of the Aponte Training Institute reflects one effort to support broader dissemination of POTT‐informed pedagogy. Yet, dissemination alone does not resolve questions of supervision preparation and sustainability. Future scholarship must articulate explicit train‐the‐trainer frameworks, supervision‐of‐supervision models, and scalable pedagogical tools capable of supporting implementation across geographically and institutionally diverse settings. At the same time, POTT may be difficult to implement without sufficient trainer preparation and institutional support. Since the model invites trainees to engage personal vulnerability in professional training contexts, supervisors and instructors must be prepared to maintain clear boundaries, emotional safety, and a non‐evaluative learning environment. Without these conditions, POTT‐informed exercises may risk becoming overly diffuse, performative, or experienced as unsafe by trainees.

Across POTT's developmental stages, the effectiveness of the approach likely depends not only on curriculum design, but also on the relational stance of trainers and supervisors. Learning environments characterized by humility, psychological safety, and non‐evaluative exploration may be especially important for supporting trainees' engagement with vulnerability, self‐reflection, and personal growth (Aponte and Kissil 2016; Hardy 2018; Hardy and Bobes 2016). Supervisors and trainers have a central role in the promotion of these learning environments. Beyond C/MFT, these developmental aims parallel longstanding concerns in psychotherapy training regarding therapist use of self, management of countertransference, immediacy, and relational presence (Hayes et al. 2018; Hill et al. 2018; Rogers 1957). Framed in this way, POTT may offer a structured model for helping clinicians engage personal experience in service of therapeutic effectiveness rather than distancing from it through purely technical performance.

### 4.4 Limitations

Several limitations should be considered when interpreting this synthesis. First, the empirical literature remains limited in scope and relies almost exclusively on qualitative methodologies derived from relatively small trainee cohorts. Consequently, conclusions regarding effectiveness, generalizability, and long‐term clinical outcomes remain preliminary. Moreover, as it was mentioned, authorship within the POTT literature is concentrated among a relatively small network of scholars connected through the Drexel University C/MFT program, including Aponte, Kissil, Niño, Zeytinoglu‐Saydam, Russon, Pennant, Apolinar Claudio, and colleagues. Although this continuity has supported conceptual depth, it also underscores the importance of broader scholarly engagement, methodological diversification, and cross‐institutional validation. Additionally, existing empirical studies have focused primarily on trainee development following participation in POTT training, with no studies to date examining client outcomes associated with therapists trained in the model. The literature also remains limited in its attention to implementation challenges, including trainer preparation, institutional support, boundary management, and trainee safety. Future empirical work might also examine whether clinicians trained to intentionally engage personal process, vulnerability, and reflexive use of self demonstrate differences in alliance development, therapist responsiveness, or clinical outcomes compared with more technique‐dominant training models. Expanding both the empirical base and the diversity of contributing voices will be essential for the continued maturation of the model.

## 5 Conclusion

This review of the POTT approach's developmental trajectories clarifies next steps for both the model and the field of systemic family therapy. In particular, the growth of POTT serves as a reminder that psychotherapy training depends on sustained attention not only to technique and theory but also to therapist presence. At a historical moment marked by rapid technological mediation, expanding artificial forms of relational interaction, and growing sociocultural complexity, the question of what constitutes authentic therapeutic presence has become increasingly urgent. The POTT framework offers one response to this question by clarifying how therapists' lived experience and attuned presence can promote change.

## Data Availability

Data sharing not applicable to this article as no datasets were generated or analyzed during the current study.
